# Using mass spectrometry to monitor drug induced changes in antigen presentation by the human leukocyte antigen

**DOI:** 10.1186/2045-7022-4-S3-P43

**Published:** 2014-07-18

**Authors:** Patricia Illing, Nicole Mifsud, Nathan Croft, Nadine Dudek, James McCluskey, Anthony Purcell

**Affiliations:** 1 Monash University, Department of Biochemistry and Molecular Biology, Australia; 2 The University of Melbourne, Department of Microbiology and Immunology, Australia

## Background

The growing list of associations between idiosyncratic adverse drug reactions and variants of the Human Leukocyte Antigen (HLA) molecule is suggestive of specific interactions between the causative drug and the associated HLA molecule. Currently, most of our understanding of how HLA molecules stimulate drug specific T cell responses comes from observing the conditions necessary for T cell stimulation (e.g. specific HLA, time for drug metabolism, constant drug presence, antigen processing). Whilst this provides indirect evidence of the presence of immunogenic ligands on the cell surface it does not determine the precise nature of the immunogenic HLA ligand(s). Using a mixture of mass spectrometry (MS) and structural biology techniques we recently defined the mode of interaction between abacavir and HLA-B*57:01. We showed how, by occupying the antigen binding cleft, abacavir changes the array of peptides bound by the HLA molecule, furnishing circulating T cells with numerous novel, potentially immunogenic, HLA-B*57:01-abacavir-peptide complexes. Regardless, there is still some debate as to whether T cell responses to abacavir are elicited by i) the binding of abacavir to HLA-peptide complexes directly at the cell surface to generate immediately available immunogenic complexes, ii) the loading of novel, abacavir enabled ligands in the endoplasmic reticulum, or iii) a mixture of both.

## Methods and results

Here we use targeted MS experiments to simultaneously monitor the binding of abacavir to, and changes in peptide presentation by, HLA-B*57:01+ cells over time, tracking a series of peptides known to bind HLA-B*57:01 under different conditions. We show that abacavir binding and changes in peptide presentation mirror the time course for maximal antigenicity of abacavir-treated HLA-B*57:01+ cell lines for abacavir responsive T cell lines, suggesting that the major part of the response is towards the binding of novel peptides.

## Conclusions

The MS techniques used here successfully tracked drug induced changes in antigen presentation by the HLA molecule and may be adapted to monitor drug induced changes in antigen presentation in other HLA-associated drug hypersensitivities.
